# GM Allotypes and COVID-19. A Pilot Study Performed on Sicilian Patients

**DOI:** 10.37825/2239-9747.1039

**Published:** 2022-12-27

**Authors:** Mattia E. Ligotti, Anna Calabrò, Giulia Accardi, Anna Aiello, Calogero Caruso, Claudia Colomba, Danilo Di Bona, Giovanni Duro, Aryan M. Namboodiri, Antonino Tuttolomondo, Janardan P. Pandey, Giuseppina Candore

**Affiliations:** a Laboratory of Immunopathology and Immunosenescence, Department of Biomedicine, Neurosciences and Advanced Diagnostics, University of Palermo, Palermo, Italy; b Department of Health Promotion, Maternal and Infant Care, Internal Medicine and Medical Specialties, University of Palermo, Palermo, Italy; c Department of Emergency and Organ Transplantation, University of Bari Aldo Moro, Bari, Italy; d Institute for Biomedical Research and Innovation, National Research Council of Italy, Palermo, Italy; e Department of Microbiology and Immunology, Medical University of South Carolina, Charleston, SC, USA

**Keywords:** ADCC, COVID-19, Immune response, Immunogenetics, γ marker

## Abstract

Several studies suggest that genetic variants that influence the onset, maintenance and resolution of the immune response might be fundamental in predicting the evolution of COVID-19. In the present paper, we analysed the distribution of GM allotypes (the genetic markers of immunoglobulin γ chains) in symptomatic and asymptomatic COVID-19 patients and in healthy controls, all born and residing in Sicily. Indeed, the role played by GM allotypes in immune responses and infection control is well known. Our findings show that the GM23 allotype is significantly reduced in healthy controls. Interestingly, in a previous study, Sicilians carrying the GM23 allotype were associated with the risk of developing a symptomatic Human Cytomegalovirus infection. However, a note of caution should be considered, due to the small sample size of patients and controls.

## 1. Introduction

Coronavirus disease 2019 (COVID-19) caused by severe acute respiratory syndrome coronavirus 2 (SARS-CoV-2) presents a variety of clinical manifestations, ranging from an asymptomatic condition to a life-threatening disease associated with cytokine storm, multi-organ and respiratory failure. The molecular mechanism underlying this variability is still under study. However, several data suggest that genetic variants that influence the onset, maintenance and resolution of the immune response may be fundamental in predicting the evolution of the disease [1,2]. Since in addition to cellular immunity also the antibody response plays a role in the control of SARS-CoV-2 infection [3], the aim of this study was to analyse the role of GM allotypes, in the context of SARS-CoV-2 infection. The term allotype refers to any genetic variant of a protein. However, in immunology it is used for hereditary antigenic determinants expressed on immunoglobulin polypeptide chains, *i.e*., the genetic markers of γ chains (GM). GM allotypes are encoded by autosomal codominant alleles on immunoglobulin heavy chain γ1, γ2 and γ3 genes [4]. It is well known the role played by immunoglobulin allotypes in immune responses and in control of infections. Indeed, allotypes of immunoglobulins have long been associated with susceptibility to infections such as malaria, Herpes simplex, Cytomegalovirus (HCMV), and Hepatitis B and C viruses (HCV) [5–11]. Allotype diversity may be related to the serum abundance of antibodies targeting the infectious agent. For example, antibody titers against the envelope E1E2 glycoprotein complex of the HCV are, in part, determined by the allotypes G1M1, G1M17, G3M5 and G3M13 (old terminology related to serum typing) and are prognostic for faster recovery [10,11].

Based on these previously findings, it is conceivable that particular GM allotypes may contribute to the generation of an efficient immune response against SARS-CoV-2 infection.

## 2. Materials and methods

In order to show if GM allotypes play a role in the pathophysiology of COVID-19, we typed the genomic DNA of 45 symptomatic patients (age range 46–88 years, 27 males and 18 females), 45 asymptomatic patients (age range 26–88 years, 27 males and 18 females), and 51 healthy controls (age range 40–70 years; 34 males and 17 females) from Sicily, for GM3/17 and GM23 +/− alleles. Real Time PCR was used in patients and controls to verify the presence and, respectively, the absence of the infection. Asymptomatic cases had no or only mild symptoms, and computed tomography (CT) imaging revealed no pneumonia. The symptomatic group had fever, respiratory symptoms, and pneumonia, as confirmed via CT imaging. Characteristics of symptomatic COVID-19 patients at the time of hospitalization are summarized in Table 1. The determination of minimum sample sizes of COVID-19 cohorts could not be performed because no previous studies were available to estimate the expected prevalence of GM in cohorts of Sicilian patients [12].

TaqMan^®^ genotyping assays were used for typing GM 3/17 and GM23 +/− alleles, as previously described [13]. IgG3 markers GM5 and GM21 were not typed, because TaqMan genotyping assays for the IgG3 allotypes are not yet available. Because of almost absolute linkage disequilibrium at GM loci within an ethnic group, subjects positive for the IgG1 allotypes GM3 and GM17 are most likely positive for the IgG3 allotypes GM5 and GM21 [9,14].

Frequencies were estimated by allele counting. Comparisons of allele carrier frequencies were made using contingency tables, analysed by the *chi*-squared test. It was not possible to type two DNA samples of symptomatic patients for GM13/17 and one DNA sample for GM23 +/− as well as five DNA samples of asymptomatic patients for GM3/17.

To respect privacy, everyone was identified with an alphanumeric code and the data were managed using a database accessible exclusively by researchers involved in the project. The study protocol, conducted in accordance with the Declaration of Helsinki and its amendments, was approved by the Ethic Committee of Palermo University Hospital.

## 3. Results and discussion

No significant variation of the frequency of the GM3 and GM17 carriers between the symptomatic and asymptomatic patients was demonstrated. Instead, the distribution of GM 23-carriers/non carriers was significantly different between the groups (P = .046375), with the lower frequency of GM23 carriers in healthy subjects, suggesting its role in the susceptibility to SARS-CoV-2 infection (Table 2).

It has been demonstrated that COVID-19-related alterations of B lymphocytes influence not only the disease severity but also the entity of long-term consequences [3]. Accordingly, antibodies are first-line molecules in adaptive immunity to fight infections. Pathogens can be neutralized or directly, via binding of the variable domains of the antibody or binding of the constant domain to receptors for the crystallizable fragment of immunoglobulins (Fc-recepor) on the immune cell membrane (see below). This results in potent inflammatory or anti-inflammatory responses that play a role in resistance/susceptibility to infection [15].

GM allotypes can affect antibody specificity and affinity by modifying the conformation of the variable region, thereby modulating the kinetic competence of the antigen binding sites [16]. The engagement profile of the Fc receptor is, in part, governed by the heavy chain isotype of the antibody. Adding an extra layer of complexity, the genes of the constant regions of the immunoglobulin heavy and light chains are polymorphic [17]. However, another important mechanism of GM gene involvement in response to SARS-CoV-2 may refer to antibody-dependent cellular cytotoxicity (ADCC), *i.e.*, the host immunosurveillance mechanism against virally infected cells. GM variation might contribute to the differences in magnitude of ADCC triggered upon ligation of the IgG Fc-receptor (Fcγ-receptor) [18]. Moreover, ADCC could explain why asymptomatic people have a higher GM23 frequency than symptomatic ones. So, we could postulate a mechanism based on neutrophil-mediated ADCC of SARS-CoV-2-infected cells. It is possible that asymptomatic individuals express a Fcγ-receptor allele which has a high affinity for GM23-expressing IgG2, resulting in potent neutrophil-mediated ADCC.

At this regard, Fcγ-receptors recognise the constant fraction of IgG mainly triggering processes like ADCC, phagocytosis or production of reactive oxygen species. Several polymorphisms have been described with consequences in their functionality that can lead to increased susceptibility to infectious diseases [19,20]. In line with the present study, several reports already demonstrated that polymorphisms of Fcγ receptors have been associated with COVID-19 outcomes [21,22].

Other mechanisms involved in resistance/susceptibility to viruses could concern a more or less efficient recognition of viral antigens by membrane-bound IgG receptors of B lymphocytes. They express different alleles that could participate as recognition structures for pathogenic epitopes on B lymphocyte membranes [18].

To the best of our knowledge, no study has evaluated the role of GM allotypes in COVID-19. In fact, GM allotypes have not been evaluated in the genome-wide association studies of COVID-19, because these determinants are not included in the commonly employed genotyping platforms [23]. Therefore, a candidate gene approach is necessary for evaluating the possible role played by GM genes in COVID-19.

In the present paper, we have analysed the distribution of GM allotypes in COVID-19 patients and healthy controls from Sicily. Data show that the GM23 allotype is significantly decreased in healthy controls. It is noteworthy that, in a previous study, Sicilians carrying the GM23 allotype were associated with the risk of developing HCMV symptomatic infection [7]. However, a note of caution must be taken into account, because of the small sample size of patients and controls. Then, we did not correct p values for multiple comparisons, but standard corrections, such as Bonferroni's, do more harm than good to sound statistical inference in biomedical research [24]. The Bonferroni's correction is, indeed, a strong killer for genetic risk factors with small effects [18].

## Figures and Tables

**Table 1:** Baseline characteristics of COVID-19 patients with symptomatic disease.

Characteristics	Symptomatic patients	
	
*Recovery time*	**Mean ± SD**	18.1 ± 1.9
	**Range day**	1–58

*Baseline haematological and haematochemical parameters*	**Mean ± SD**	

WBC (10^3^μl)	8.9 ± 6.2	
Lymphocyte (10^3^μl)	0.8 ± 0.46	
Lymphocyte (%)	12.2 ± 8.3	
Monocytes (10^3^μl)	0.5 ± 0.33	
Monocytes (%)	7 ± 4.3	
Red blood cells (10^6^μl)	4.5 ± 0.7	
Hb (g/dl)	13.2 ± 1.5	
Creatinine (mg/dl)	1.09 ± 0.57	
LDH (UL)	339.02 ± 139.5	
PCR (mg/L)	82 ± 65.45	
IL-6 (pg/mL)	42 ± 44.5	
D-dimer (ng/mL FEU)	6761.3 ± 17835.4	
Troponin (ng/L, 99th percentile)*	34.3 ± 45.5	

*Baseline diseases*	**N (%)**	

Hypertension	12 (27)	
Diabetes	9 (20.4)	
Dyslipidemia	1 (0.9)	
Chronic obstructive pulmonary disease	9 (20.4)	
Cardiovascular disease	13 (29.5)	
Others♦	29 (66)	

*Baseline symptomatology*	**N (%)**	

Respiratory failure	44 (100)	
ARDS	5 (11.4)	

FEU, fibrinogen equivalent unit; SD, standard deviation.

* Data on troponin levels are missing in 10 COVID-19 patients.

♦ Other baseline diseases include chronic kidney disease, obesity, HCV-related cirrhosis, Crohn's disease, Familial adenomatous polyposis, Favism.

**Table 2:** Distribution of GM3,17,23 carriers in symptomatic and asymptomatic patients and healthy controls.

Genotypes	Symptomatic patients	Asymptomatic Patients	Healthy controls
		
	N (%)	N (%)	N (%)
GM3 carriers	66 (76.74)	65 (81.25)	70 (68.62)
GM17 carriers	20 (23.26)	15 (18.75)	32 (31.37)
GM23 carriers	40 (45.45)	52 (57.78)	41 (40.20)
GM23 non carriers	48 (54.55)	38 (42.22)	61 (59.80)

*P* = .046375 for GM23 carriers/non carriers.
